# When Hemolysis Misleads: Implications for the Clinical Interpretation of Enzyme Activities in Pediatric Samples

**DOI:** 10.3390/medicina62091724

**Published:** 2026-09-07

**Authors:** Dejan Dobrijević, Kristian Pastor

**Affiliations:** 1 Institute for Children and Youth Health Care of Vojvodina, 21000 Novi Sad, Serbia; 2 Faculty of Medicine, University of Novi Sad, 21000 Novi Sad, Serbia; 3 Faculty of Technology Novi Sad, University of Novi Sad, 21000 Novi Sad, Serbia

**Keywords:** hemolysis, plasma enzymes, laboratory interference, preanalytical error, biochemical analysis

## Abstract

*Background and Objectives*: Hemolysis is one of the most frequent preanalytical interferences in clinical laboratory practice. This laboratory-based experimental study evaluated the effect of different degrees of hemolysis on plasma enzyme activities in pediatric blood samples. *Materials and Methods*: Twenty-six venous blood samples collected in lithium-heparin tubes were gently homogenized and divided into four equal whole-blood aliquots. One aliquot served as the untreated reference, while the remaining three aliquots were subjected to two, four, or eight aspiration–reinjection cycles through an 18-gauge needle to induce increasing degrees of erythrocyte lysis. All four aliquots were subsequently centrifuged under identical conditions and classified according to the semiquantitative hemolysis component of the analyzer-generated LIH index as non-hemolyzed, H1, H2, H3, H4, or H5. Activities of alpha-amylase, alkaline phosphatase, alanine aminotransferase, aspartate aminotransferase, gamma-glutamyl transferase, creatine kinase, and lactate dehydrogenase, as well as potassium concentration, were measured using the same DxC 700 AU automated clinical chemistry analyzer. Statistical analysis included 26 samples and was performed using the Wilcoxon signed-rank test. *Results*: Hemolysis caused a progressive increase in lactate dehydrogenase, aspartate aminotransferase, creatine kinase, alanine aminotransferase, and potassium, with the strongest effects observed for lactate dehydrogenase and aspartate aminotransferase. Alpha-amylase and alkaline phosphatase showed a decreasing trend, while gamma-glutamyl transferase showed variable behavior across hemolysis groups. *Conclusions*: The findings confirm that even low degrees of hemolysis can substantially alter selected biochemical results, especially lactate dehydrogenase, aspartate aminotransferase, and creatine kinase. Laboratory reports from hemolyzed pediatric samples should therefore be interpreted with caution to reduce the risk of clinically misleading results.

## 1. Introduction

Hemolysis is defined as the disruption of the erythrocyte membrane, resulting in the release of intracellular components into the surrounding plasma or serum [1,2]. It may occur in vivo as part of pathological processes such as hemolytic anemia, sepsis, parasitic infections, or hemoglobinopathies, or in vitro during blood collection, transportation, processing, storage, or analysis [2,3]. Distinguishing between these two forms is clinically important. In vivo hemolysis reflects an underlying pathological condition and may itself require diagnostic evaluation, whereas in vitro hemolysis does not represent the patient’s physiological state and is generally considered a potentially preventable preanalytical error [2,4,5].

Hemolysis is one of the most frequent preanalytical problems encountered in clinical laboratory practice and an important cause of unsuitable or rejected blood specimens [2,5,6,7]. Most hemolyzed samples result from events occurring before analysis, particularly during venipuncture and specimen handling [5,6,7]. Factors associated with in vitro hemolysis include difficult blood collection, prolonged tourniquet application, incomplete drying of the skin disinfectant, use of inappropriate needles, forceful aspiration or transfer of blood with a syringe, collection through an intravenous catheter, vigorous tube mixing, excessive centrifugation, and inappropriate temperature conditions during transport or storage [1,3,5,8]. Since many of these factors can be reduced through standardized procedures, appropriate equipment, staff education, and monitoring of specimen quality, the frequency of hemolyzed samples is also regarded as an indicator of preanalytical performance [5,6,7].

The effect of hemolysis on laboratory results is complex and depends on the analyte, the degree of erythrocyte damage, and the analytical method used [2,3,4]. Intracellular release may directly increase the measured concentration or activity of substances that are present in considerably higher amounts in erythrocytes than in plasma. This is particularly relevant for potassium, lactate dehydrogenase (LDH), aspartate aminotransferase (AST), and, to a lesser extent, alanine aminotransferase (ALT) [2,3]. Conversely, hemolysis may decrease the measured concentration or activity of analytes that are present at lower concentrations in erythrocytes or are affected by dilutional and chemical processes. Reported examples include gamma-glutamyl transferase (GGT), alkaline phosphatase (ALP), sodium, chloride, albumin, glucose, and bilirubin [2]. For bilirubin, this refers to method-dependent analytical interference in an already collected specimen and should not be confused with the increase in bilirubin that may accompany in vivo hemolysis.

In addition to the direct release or dilution of analytes, free hemoglobin and other intracellular components may interfere with laboratory measurements through several mechanisms [2,3]. Hemoglobin may cause spectrophotometric interference because of its light absorption characteristics, while released intracellular substances may interact with assay reagents, compete with assay substrates, or alter reaction kinetics [2,3]. Proteolytic substances released from damaged cells may also affect the stability of some proteins and enzymes [2]. Consequently, the direction and magnitude of interference may differ among analytes and analytical systems, and even a visually mild degree of hemolysis may substantially alter selected biochemical results [2,4].

Hemolysis-associated interference may have clinically important consequences. Falsely increased enzyme activities may be interpreted as evidence of hepatic, muscular, cardiac, or generalized tissue injury, while an artifactual increase in potassium may be mistaken for true hyperkalemia [2,3]. Conversely, falsely decreased results may conceal an existing abnormality or lead to underestimation of disease severity. Such findings may result in unnecessary repeat testing, additional diagnostic procedures, inappropriate treatment, delayed clinical decisions, or failure to recognize a genuine pathological process [3,5]. Clinical interpretation becomes particularly challenging when the altered result appears biologically plausible and the degree of hemolysis is not immediately apparent.

The management of hemolyzed specimens therefore requires a balance between analytical reliability and the clinical consequences of rejecting a sample [2]. Depending on the analyte and the degree of interference, laboratories may request recollection, suppress the affected result, or release it with an appropriate interpretative comment [2]. However, repeat collection may not always be simple or clinically desirable, particularly in pediatric and emergency settings. Blood collection in children is often technically demanding because of smaller veins, patient movement, anxiety, and limited cooperation [1,9]. Repeat venipuncture may cause additional pain and distress, delay diagnosis and treatment, and further limit the available blood volume [1,9].

Pediatric specimens may therefore present a particular laboratory challenge. On the one hand, reporting substantially affected results may expose the child to diagnostic or therapeutic error. On the other hand, automatic rejection of every hemolyzed specimen may lead to repeated sampling and delays in clinical management. Preanalytical errors are common in pediatric laboratory practice, especially when sample collection is technically difficult or when only a limited specimen volume is available [1,9,10,11]. Nevertheless, relatively few studies have specifically evaluated the effect of different degrees of hemolysis on biochemical results obtained from pediatric blood samples [1,10,11]. In this context, the incremental contribution of the present study is the paired evaluation of graded hemolysis in pediatric lithium-heparin plasma using a single analytical system, providing method- and matrix-specific data that may support analyte-specific interpretation and recollection decisions in pediatric laboratory practice.

Therefore, the aim of this study was to quantify the direction and magnitude of hemolysis-associated bias in selected enzyme activities and potassium concentration in experimentally hemolyzed pediatric heparin plasma samples. A secondary aim was to determine whether measurable interference was already present at lower hemolysis-index categories.

## 2. Materials and Methods

### 2.1. Study Design and Sample Selection

This laboratory-based experimental study was conducted at the Department of Laboratory Diagnostics, Institute for Children and Youth Health Care of Vojvodina, Novi Sad, Serbia. Venous blood specimens collected from pediatric patients during routine laboratory testing between October and November 2024 were considered for inclusion.

Blood collection was performed by trained healthcare professionals using evacuated tubes containing lithium heparin as the anticoagulant and without a separator gel (Becton Dickinson, Franklin Lakes, NJ, USA). A total of 26 specimens that met the predefined eligibility criteria were included in the study. No formal a priori sample-size calculation was performed. The sample size was feasibility-based and reflected the number of eligible pediatric specimens available during the predefined study period that met the volume and quality requirements for the four-aliquot experimental protocol.

Specimens were eligible if they contained at least 2.0 mL of venous blood, showed no visible evidence of hemolysis, and had no marked lipemia, icterus, clot formation, or other abnormal appearance. Final inclusion required the untreated reference aliquot to have an analyzer-generated hemolysis result below H1. Specimens were excluded in cases of insufficient volume, collection in an inappropriate tube, visible hemolysis before experimental processing, clot formation, marked lipemia or icterus, or a baseline hemolysis category of H1 or higher.

### 2.2. Sample Preparation and Experimental Induction of Hemolysis

Immediately after collection, each lithium-heparin whole-blood specimen was gently homogenized by eight complete tube inversions. The specimen was then divided into four equal aliquots of 500 µL in individually labeled capped centrifuge tubes.

One aliquot was designated as the untreated reference condition and was not subjected to aspiration–reinjection through a needle. The remaining three aliquots were mechanically stressed by repeatedly aspirating and reinjecting the whole blood through an 18-gauge needle attached to a 10 mL syringe. The procedure was performed for two, four, or eight aspiration–reinjection cycles in the respective aliquots.

The aliquots were processed sequentially according to a predefined protocol, and the interval between aliquot preparation and centrifugation was kept identical for all four conditions. After mechanical treatment, the untreated and mechanically stressed aliquots were centrifuged simultaneously for 10 min at 2000× *g* at room temperature.

Following centrifugation, plasma was carefully transferred from each aliquot into a separate analyzer insert cup without disturbing the cellular layer. The untreated reference and all mechanically treated aliquots were analyzed during the same analytical run.

The number of aspiration–reinjection cycles was used only to generate increasing degrees of erythrocyte damage. The final hemolysis category was assigned according to the analyzer-generated hemolysis component of the LIH index rather than according to the number of mechanical stress cycles.

### 2.3. Biochemical Measurements

The activities of alpha-amylase (AMY), alkaline phosphatase (ALP), alanine aminotransferase (ALT), aspartate aminotransferase (AST), gamma-glutamyl transferase (GGT), creatine kinase (CK), and lactate dehydrogenase (LDH) were measured in the untreated reference and mechanically stressed lithium-heparin plasma aliquots using a DxC 700 AU automated clinical chemistry analyzer (Beckman Coulter, Fullerton, CA, USA). All four aliquots originating from the same specimen were analyzed within the same analytical run using the same reagent lots, calibration status, and quality-control conditions.

AMY and GGT activities were determined using methods based on the recommendations of the International Federation of Clinical Chemistry and Laboratory Medicine. ALP activity was measured using the p-nitrophenyl phosphate method, ALT and AST activities using ultraviolet kinetic methods, CK activity using an IFCC-recommended method, and LDH activity using an ultraviolet method. Enzyme activities were expressed as microkatals per liter (µkat/L). For conversion to conventional enzymatic units, 1 µkat/L corresponds to 60 U/L.

Potassium concentration was measured using an indirect ion-selective electrode method and expressed in mmol/L. Potassium was included as an additional biochemical indicator of erythrocyte disruption because its concentration is expected to increase following the release of intracellular erythrocyte contents.

### 2.4. Classification of Hemolysis

Sample hemolysis was assessed using the hemolysis component of the analyzer-generated lipemia–icterus–hemolysis (LIH) index on the DxC 700 AU automated clinical chemistry analyzer (Beckman Coulter, Fullerton, CA, USA). The hemolysis index was reported semiquantitatively as categorical levels corresponding to manufacturer-defined approximate free hemoglobin intervals, rather than as an exact continuous free hemoglobin concentration. Hemolyzed aliquots were classified as H1 (50–99 mg/dL), H2 (100–199 mg/dL), H3 (200–299 mg/dL), H4 (300–500 mg/dL), or H5 (>500 mg/dL).

Aliquots with an analyzer-generated hemolysis result below H1, corresponding to an estimated free hemoglobin concentration below 50 mg/dL, were classified as non-hemolyzed and served as the patient-specific reference condition for each biochemical parameter. Each hemolyzed result was therefore compared with the corresponding non-hemolyzed aliquot obtained from the same original specimen.

Because hemolysis was induced manually, the same number of specimens was not obtained in each hemolysis category. Consequently, the number of available paired comparisons varied among the H1–H5 categories.

The untreated aliquot was classified as the patient-specific reference condition if its analyzer-generated hemolysis result was below H1, corresponding to an estimated free hemoglobin concentration below 50 mg/dL. Original specimens for which the untreated aliquot was classified as H1 or higher were excluded from further analysis.

Each mechanically treated aliquot was compared with the corresponding untreated aliquot prepared from the same original specimen. Because all aliquots were obtained from the same homogenized whole-blood specimen and were subjected to identical storage, centrifugation, and analytical conditions, the paired difference was considered to predominantly reflect the effect of mechanically induced hemolysis.

Mechanically treated aliquots were classified as H1 (50–99 mg/dL), H2 (100–199 mg/dL), H3 (200–299 mg/dL), H4 (300–500 mg/dL), or H5 (>500 mg/dL). Treated aliquots that remained below H1 were not included in the category-specific hemolysis analyses.

If two mechanically treated aliquots originating from the same original specimen were assigned to the same hemolysis category, their biochemical results were averaged before statistical analysis. Consequently, each original specimen contributed no more than one paired observation to an individual hemolysis category.

### 2.5. Statistical Analysis

Statistical analysis was performed using data obtained from 26 original blood specimens. The original specimen, rather than the individual aliquot, was treated as the statistical unit. Results were processed using Microsoft Excel 365 (Microsoft Corporation, Redmond, WA, USA) and jamovi 2.6.44 (The jamovi Project, Sydney, Australia).

For each hemolysis category, the result obtained from the mechanically treated aliquot was compared with the corresponding untreated reference result from the same original specimen. If more than one treated aliquot from the same specimen was classified within the same hemolysis category, the treated results were averaged before the paired analysis. Differences between untreated and hemolyzed measurements were evaluated using the Wilcoxon signed-rank test. Because different subsets of original specimens contributed to the H1–H5 categories and no specimen had observations in all five hemolysis categories, the data did not constitute a complete repeated-measures dataset across categories. Separate paired comparisons were performed for each hemolysis category. Adjustment for multiple comparisons was performed separately within each analyte across the five hemolysis-category comparisons using the Holm procedure. Each analyte was considered a separate hypothesis family because the study was designed to evaluate analyte-specific effects of hemolysis; therefore, adjustment was performed across the five hemolysis-category comparisons within each analyte rather than across all analytes jointly.

The percentage change relative to the corresponding non-hemolyzed aliquot was calculated as follows:

Percentage change = [(hemolyzed result − non-hemolyzed result)/non-hemolyzed result] × 100.

Positive values indicated an increase, whereas negative values indicated a decrease relative to the non-hemolyzed reference condition. Only aliquots in which mechanical treatment produced a hemolysis category of H1 or higher were included in the corresponding paired analysis. A two-sided *p* value below 0.05 was considered statistically significant.

### 2.6. Ethics Statement

The study protocol was approved by the Ethics Committee of the Institute for Children and Youth Health Care of Vojvodina, Novi Sad, Serbia (Approval No. 2461-3, 16 May 2024). All study procedures were conducted in accordance with the principles of the Declaration of Helsinki.

## 3. Results

### 3.1. Distribution of Samples Across Hemolysis Categories

A total of 26 original pediatric blood samples were included in the study. Each specimen was divided into one untreated reference aliquot and three mechanically treated aliquots, yielding 104 aliquots in total, including 26 untreated and 78 treated aliquots. All aliquots were subjected to identical storage, centrifugation, and analytical conditions.

Of the 78 mechanically treated aliquots, 65 were classified as H1 or higher and were included in the category-specific analyses. The remaining 13 treated aliquots had an analyzer-generated hemolysis result below H1 and were not included as hemolyzed samples.

Following classification, 22 paired observations were available for H1, 14 for H2, 10 for H3, 10 for H4, and 9 for H5. Individual original specimens could contribute observations to more than one hemolysis category. However, each specimen contributed no more than one paired observation within an individual category. In the present dataset, no original specimen had more than one mechanically treated aliquot assigned to the same hemolysis category; therefore, averaging of treated results was not required.

The mean percentage bias in the investigated biochemical parameters relative to the corresponding untreated reference aliquots is presented in Table 1.

### 3.2. Positive Interference in Enzyme Activities and Potassium Concentration

Hemolysis produced predominantly positive interference in LDH, AST, CK, ALT, and potassium measurements. The magnitude of the effect generally increased with the degree of hemolysis, although the pattern was not strictly monotonic for all analytes.

LDH was the analyte most strongly affected by hemolysis. Mean LDH activity increased by 90.44% in H1, 150.17% in H2, 215.71% in H3, 319.92% in H4, and 480.28% in H5. Among the 22 H1 pairs, median LDH activity increased from 3.35 µkat/L (IQR, 2.75–4.05) in the untreated reference aliquots to 6.10 µkat/L (IQR, 5.15–7.55) in the corresponding H1 aliquots. Among the nine H5 pairs, median LDH activity increased from 3.55 µkat/L (IQR, 3.00–4.30) to 19.10 µkat/L (IQR, 15.60–23.90). Differences from the corresponding untreated reference aliquots were statistically significant in all hemolysis categories and remained significant after correction for multiple comparisons.

AST showed the second largest increase. Mean AST activity increased by 37.94% in H1, 68.97% in H2, 85.55% in H3, 125.84% in H4, and 234.27% in H5. Among the H1 pairs, median AST activity increased from 0.44 µkat/L (IQR, 0.35–0.55) in the untreated reference aliquots to 0.60 µkat/L (IQR, 0.47–0.75) in the corresponding hemolyzed aliquots. Among the H5 pairs, median AST activity increased from 0.47 µkat/L (IQR, 0.39–0.59) to 1.52 µkat/L (IQR, 1.18–1.93). All comparisons remained statistically significant after Holm correction.

CK showed an overall increase with the severity of hemolysis, although the pattern was not strictly monotonic. Mean CK activity increased by 7.05% in H1, 24.70% in H2, 16.00% in H3, 34.19% in H4, and 48.66% in H5. The lower bias in H3 than in H2 indicated some variability between categories. Nevertheless, CK activity differed significantly from the corresponding non-hemolyzed values in all categories, and the differences remained significant after adjustment for multiple testing.

ALT was less affected at lower degrees of hemolysis. Mean ALT activity increased by 3.44% in H1, 9.17% in H2, 10.17% in H3, 23.68% in H4, and 35.46% in H5. The unadjusted differences were statistically significant in H1, H2, H4, and H5, but not in H3. Following Holm correction, the differences remained significant in H1, H4, and H5, whereas the H2 comparison no longer reached statistical significance.

Potassium concentration increased substantially even at the lowest degree of hemolysis. Mean percentage bias was 22.46% in H1, 33.26% in H2, 34.70% in H3, 54.26% in H4, and 70.73% in H5. Among the 22 H1 pairs, median potassium concentration increased from 4.08 mmol/L (IQR, 3.88–4.33) in the untreated reference aliquots to 4.96 mmol/L (IQR, 4.66–5.40) in the corresponding H1 aliquots. Among the nine H5 pairs, median potassium concentration increased from 4.12 mmol/L (IQR, 3.94–4.38) to 7.05 mmol/L (IQR, 6.48–7.72). All comparisons remained statistically significant after correction for multiple comparisons.

**Figure 1:**
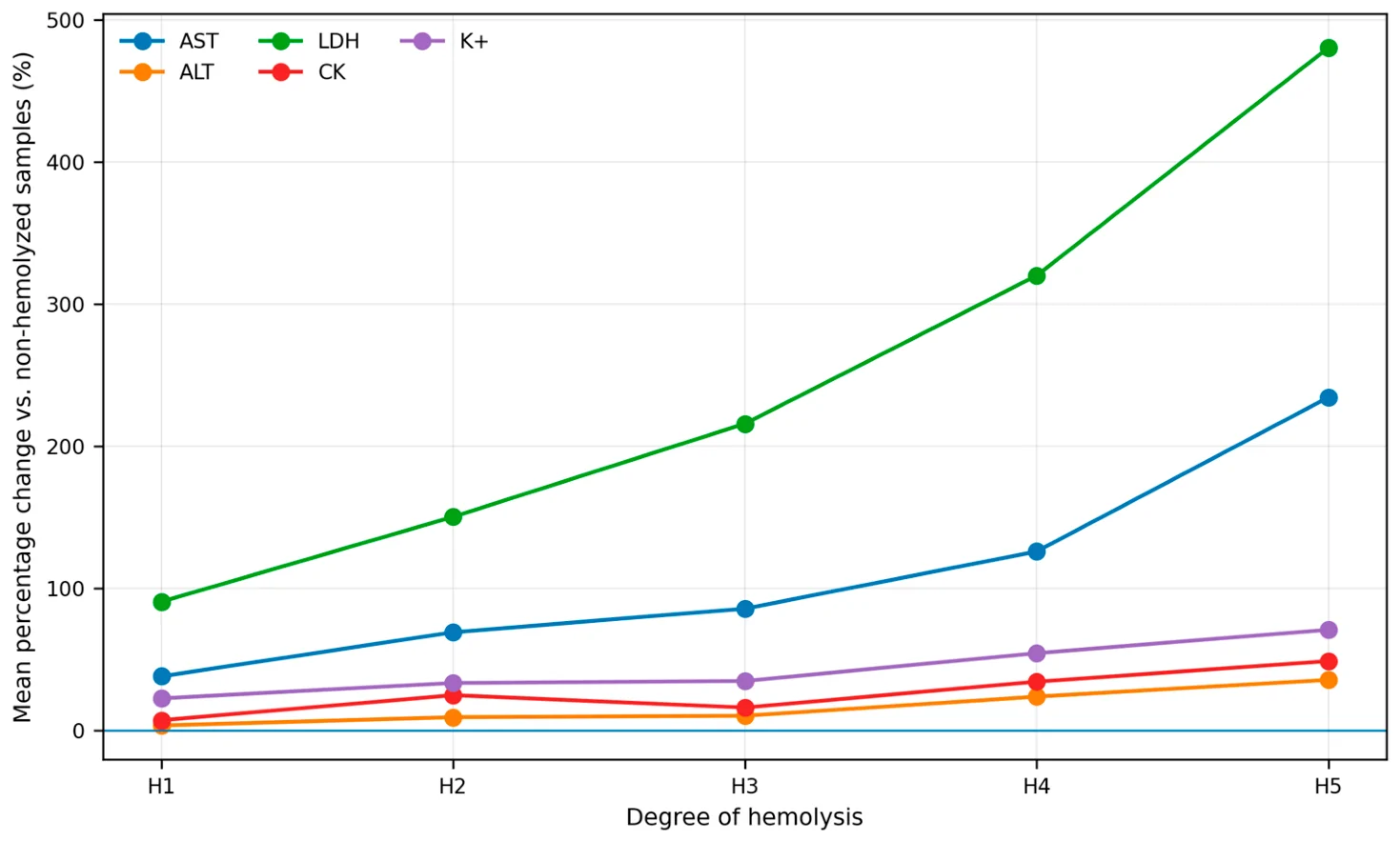
Mean percentage bias in potassium concentration and in the activities of AST, ALT, LDH, and CK relative to the corresponding untreated reference aliquots from the same original specimens across hemolysis categories H1–H5. Positive values indicate increased measurements compared with the non-hemolyzed reference condition.

The mean percentage changes in potassium concentration and in the activities of enzymes showing predominantly positive interference are illustrated in Figure 1.

### 3.3. Negative or Inconsistent Interference in Enzyme Activities

AMY and ALP showed progressively greater negative bias with increasing hemolysis, whereas GGT demonstrated an inconsistent pattern.

Mean AMY activity decreased by 1.99% in H1, 4.35% in H2, 5.03% in H3, 6.55% in H4, and 8.49% in H5. The unadjusted differences were statistically significant from H2 to H5. However, following Holm correction, only the decrease observed in H4 remained statistically significant. Despite statistical significance in selected categories, the magnitude of the AMY bias remained substantially smaller than that observed for LDH, AST, CK, or potassium.

ALP activity decreased by 1.25% in H1, 3.28% in H2, 3.75% in H3, 5.38% in H4, and 8.99% in H5. The difference reached statistical significance only in H4. Based on the multiple-comparison analysis, the H4 difference remained marginally significant after Holm correction, while all other comparisons were non-significant.

GGT showed no consistent association with the severity of hemolysis. Mean GGT activity decreased by 0.95% in H1, 7.09% in H2, and 2.67% in H3, increased by 2.24% in H4, and decreased by 9.66% in H5. Although the unadjusted comparisons were statistically significant in H3 and H5, neither difference remained significant after Holm correction. These results indicate considerable variability and do not support a clear concentration-dependent effect of hemolysis on GGT activity (Table 2).

**Figure 2:**
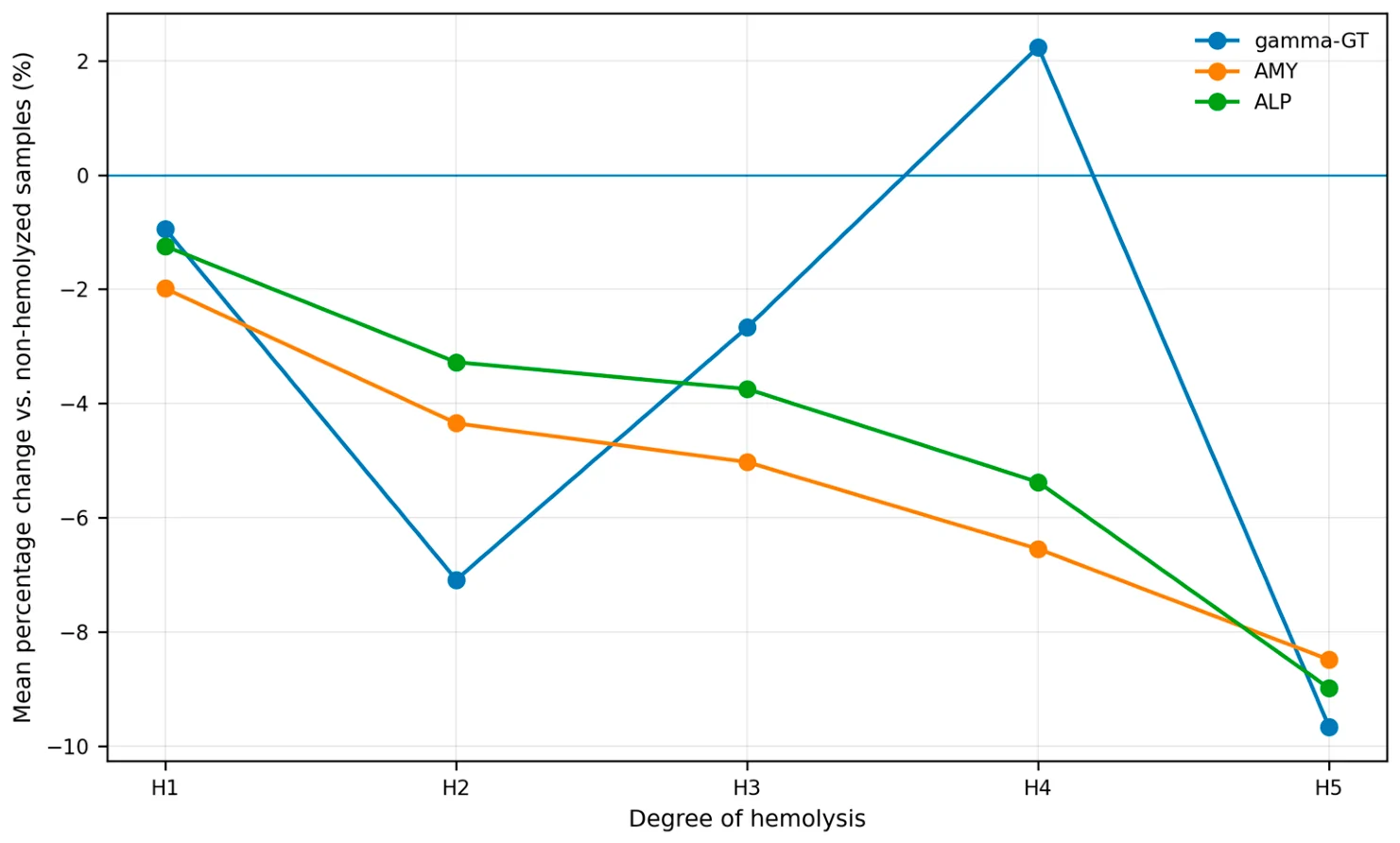
Mean percentage bias in AMY, ALP, and GGT activities relative to the corresponding untreated reference aliquots across hemolysis categories H1–H5. Negative values indicate decreased enzyme activity. GGT showed an inconsistent direction of interference across categories.

The mean percentage changes in AMY, ALP, and GGT activities are illustrated in Figure 2.

The H1 and H5 paired sets were not identical; therefore, each hemolysis category is presented with its own corresponding untreated reference values. Mean percentage bias in Table 1 was calculated from individual paired percentage changes and therefore does not necessarily equal the percentage change calculated from the group medians presented in this table (Table 3).

### 3.4. Results After Correction for Multiple Comparisons

Following the Holm correction, the increases in LDH, AST, CK, and potassium remained statistically significant across all hemolysis categories. For ALT, significant differences remained in H1, H4, and H5, whereas the H2 difference no longer reached statistical significance and the H3 comparison remained non-significant.

For analytes showing negative or variable interference, only the AMY and ALP differences observed in H4 remained statistically significant after adjustment. The differences observed for GGT did not remain significant after correction for multiple comparisons.

The results demonstrated that LDH, AST, and potassium were strongly affected even at the lowest degree of hemolysis. CK showed a smaller but statistically robust positive interference, whereas ALT became more substantially affected at higher hemolysis categories. The decreases in AMY and ALP were comparatively modest, while the effect on GGT was inconsistent.

## 4. Discussion

The present study demonstrated that experimentally induced hemolysis produced marked and analyte-dependent changes in enzyme activities and potassium concentration in pediatric heparin plasma samples. The strongest positive interference was observed for LDH and AST, followed by potassium and CK, whereas ALT showed smaller increases, particularly at lower degrees of hemolysis. In contrast, AMY and ALP showed progressively greater negative bias, while GGT did not demonstrate a consistent relationship with hemolysis severity. Importantly, substantial changes in several analytes were already observed in the lowest hemolysis category, indicating that mild hemolysis should not automatically be regarded as analytically or clinically negligible. Because the untreated and mechanically stressed aliquots were prepared from the same homogenized whole-blood specimen and were subjected to identical centrifugation and analytical conditions, the observed paired differences were unlikely to result from systematic differences in sample processing.

Potassium concentration increased significantly across all hemolysis categories, supporting the successful experimental induction of erythrocyte lysis. This finding was expected because potassium is present at a substantially higher concentration within erythrocytes than in plasma and is released following disruption of the erythrocyte membrane [12]. However, the magnitude of the change observed in H1 is clinically important. A mean increase of 22.46% could convert a potassium result within the reference interval into an apparently increased value. Among the H1 pairs, the observed median potassium concentration increased from 4.08 to 4.96 mmol/L. For illustration, applying the mean H1 bias of 22.46% to an initial potassium concentration of 4.2 mmol/L would yield a result of approximately 5.1 mmol/L. This example demonstrates how mild hemolysis could produce pseudohyperkalemia and lead to unnecessary repeat testing, electrocardiographic evaluation, interruption of medication, or inappropriate treatment if the interference is not recognized. Conversely, attributing every increased potassium result to hemolysis may also be unsafe because true hyperkalemia can coexist with specimen hemolysis. Therefore, the laboratory result must be interpreted together with the hemolysis index, previous potassium measurements, renal function, clinical presentation, and, when necessary, a promptly recollected sample [2,3,12].

LDH was the analyte most strongly affected by hemolysis, with a mean increase of 90.44% already observed in H1 and a progressive rise to 480.28% in H5. AST showed a similar pattern, increasing by 37.94% in H1 and 234.27% in H5. These findings are consistent with the high intracellular activities of LDH and AST in erythrocytes and with previous studies reporting substantial positive interference across different analytical platforms [4,13,14,15]. Clinically, falsely increased LDH may be interpreted as evidence of tissue injury, hemolysis occurring within the patient, hepatic disease, muscle injury, or another pathological process associated with cellular destruction. Similarly, an artificial increase in AST may be attributed to hepatic or muscular injury, particularly when AST is interpreted without considering the degree of sample hemolysis. In pediatric patients, such findings could prompt unnecessary repetition of liver function tests, additional measurement of muscle markers, imaging, specialist consultation, or investigation for an underlying systemic disorder.

The disproportionate effect of hemolysis on AST relative to ALT may also alter the apparent relationship between the two aminotransferases. Because AST increased substantially even in H1, whereas the ALT increase was only 3.44%, hemolysis could produce an apparently AST-predominant aminotransferase pattern. This difference is biologically plausible because erythrocytes contain substantially greater AST activity relative to plasma, whereas their contribution to ALT activity is smaller. Consequently, erythrocyte lysis affects AST more strongly than ALT, although AST should not be regarded as a specific marker of specimen hemolysis because increased AST may also have a genuine biological origin. Such a pattern may be incorrectly interpreted as having a biological origin rather than being partly or predominantly preanalytical. Therefore, AST and LDH results from hemolyzed samples should not be interpreted in isolation, especially when they are inconsistent with ALT, bilirubin, GGT, CK, clinical findings, or previous laboratory results.

CK activity showed predominantly positive interference, although the increase was smaller than that observed for LDH and AST and was not strictly monotonic across the hemolysis categories. The mean bias ranged from 7.05% in H1 to 48.66% in H5. This effect may be partly explained by the release of erythrocyte adenylate kinase, which can interfere with some CK assays and produce falsely increased activity [13,16,17,18]. In clinical practice, an artificial increase in CK may exaggerate the apparent degree of skeletal muscle injury or contribute to an incorrect suspicion of myositis, rhabdomyolysis, trauma-related muscle damage, or another muscular disorder. The clinical consequences are more likely when the original CK concentration is close to a diagnostic or action threshold. Nevertheless, because true muscle injury may coexist with a hemolyzed specimen, the finding should not be automatically dismissed. Recollection is particularly appropriate when CK is unexpectedly increased or when the result could influence further diagnostic investigation or treatment.

ALT showed relatively small changes at low degrees of hemolysis and more pronounced increases in H4 and H5. The absence of statistical significance in H3, despite a mean increase similar to that observed in H2, indicates variability and the limited precision associated with the smaller number of observations. Previous studies have also reported method-dependent effects of hemolysis on ALT [4,17,18]. From a clinical perspective, mild hemolysis is less likely to substantially alter ALT interpretation than AST or LDH, but marked hemolysis may still exaggerate an existing elevation or shift a borderline ALT value above the reference limit. The combination of a large AST increase and a smaller ALT increase is likely to be more clinically misleading than the ALT change alone.

AMY and ALP showed negative interference, with progressively greater decreases as hemolysis increased. Although the magnitude of the mean bias remained below 10% even in H5, negative interference has a different potential clinical consequence from positive interference: it may partially conceal a genuine increase. A decreased AMY result reduced the apparent magnitude of an enzyme elevation in a patient being evaluated for pancreatic or salivary disease. Similarly, a negative ALP bias could lead to underestimation of an increased activity associated with hepatobiliary or bone-related conditions. This consideration is particularly relevant in children because ALP activity is strongly influenced by age and physiological bone growth, and interpretation already requires age-appropriate reference intervals. Nevertheless, the observed mean decreases were relatively modest, and the present study did not establish whether they exceeded clinically acceptable analytical limits. Their statistical significance should therefore not be interpreted as direct evidence of clinically meaningful interference.

GGT showed an inconsistent pattern, with decreases in four categories and a slight increase in H4. This finding does not support a predictable concentration-dependent effect of hemolysis on GGT activity. Consequently, it would not be appropriate to apply a correction factor or assume a uniform direction of interference. The isolated statistically significant changes may reflect analytical variability, the limited sample size, or the simultaneous effects of dilutional, optical, and chemical interference [2,17,19]. These mechanisms may also contribute to the negative bias observed for AMY and ALP, which therefore cannot be explained simply by differences in enzyme activity between erythrocytes and plasma. Because the present study did not include mechanistic interference experiments, the relative contributions of optical or chemical interference, dilutional effects, or possible enzyme inactivation cannot be determined from our data. When GGT is clinically important, particularly in the assessment of a suspected cholestatic or hepatobiliary pattern, a new specimen should be considered if hemolysis is substantial or if the result is discordant with ALP, bilirubin, or other clinical findings.

The present findings also have implications for laboratory reporting policies. The use of lithium-heparin plasma, which is an accepted specimen type for the investigated analytical methods, reduces the likelihood that the observed changes were caused by anticoagulant-specific interference rather than by hemolysis itself. A single rejection rule applied equally to all biochemical tests is unlikely to be appropriate because the magnitude of hemolysis-associated interference differed substantially among analytes. LDH, AST, potassium, and CK were affected even at relatively low degrees of hemolysis, whereas AMY and ALP showed smaller negative changes and GGT showed no consistent pattern. Laboratories should therefore use analyte-specific and method-specific criteria when deciding whether to release, suppress, or comment on a result. The biochemical pattern observed in this study should not be used to distinguish in vivo from in vitro hemolysis; its intended relevance is the analyte-specific assessment of results from specimens already identified as hemolyzed. Correction formulas are generally not recommended because interference varies according to the analytical method, reagent formulation, specimen matrix, and degree of hemolysis [2]. When recollection is not possible, the report should clearly indicate the presence of hemolysis and identify results that may be falsely increased, falsely decreased, or unreliable. Artificial intelligence may also support future management of hemolyzed specimens by integrating specimen-quality indices with analyte-specific information, previous laboratory results, and other available data to assist laboratory decision-making. However, such systems would require local validation, continuous performance monitoring, and appropriate human oversight before implementation in routine practice [20,21].

These considerations are especially relevant in pediatric practice. Blood collection in children may be technically challenging because of small veins, movement, fear, and limited cooperation, while repeated venipuncture may cause additional pain, distress, diagnostic delay, and blood loss [19,22]. Automatic rejection of every mildly hemolyzed specimen may therefore not always be clinically desirable. However, releasing substantially biased results without an appropriate warning may lead to unnecessary investigation or treatment. A practical approach should consider the affected analyte, the degree of hemolysis, the magnitude and plausibility of the result, previous laboratory findings, and the urgency of the clinical situation. Based on the present results, recollection should be strongly considered when hemolyzed samples are used for the assessment of potassium, LDH, AST, or CK and the result could influence an immediate clinical decision.

This study has several limitations. The number of original samples was relatively small, and the number of available paired observations differed among hemolysis categories. Accordingly, non-significant findings, particularly in the smaller H3–H5 groups, should be interpreted cautiously because of the limited precision of these comparisons. Because the same original specimen could contribute to more than one hemolysis category, the category-specific comparisons within an analyte were not fully independent. This repeated-measures dependency was not explicitly modeled in the present analysis and could be addressed in future studies using an appropriate repeated-measures or mixed-effects approach. Hemolysis was assessed semiquantitatively using analyzer-generated LIH categories rather than by continuous measurement of free hemoglobin concentration, which limited detailed evaluation of the concentration–response relationship. Consequently, the present study was not designed to derive quantitative interference thresholds for result reporting or sample rejection. Mechanically induced hemolysis may not reproduce all forms of in vitro hemolysis encountered during routine venipuncture, transportation, and specimen processing. The 18-gauge aspiration–reinjection procedure was used as a standardized method to generate graded hemolysis and was not intended to reproduce a specific pediatric phlebotomy technique. Hemolysis associated with smaller-gauge needles or difficult pediatric blood collection may differ in its mechanism and magnitude; therefore, the present bias estimates should primarily be interpreted in relation to the measured hemolysis category and the analytical system used. In addition, the use of lithium-heparin plasma and a single analytical platform limits direct generalizability to serum samples, other anticoagulants, and other analyzer–reagent combinations. Therefore, the findings should be interpreted as method- and matrix-specific.

Future studies should include larger pediatric sample sets, quantitative free hemoglobin measurements, predefined allowable-bias criteria, and verification using routinely employed serum and lithium-heparin plasma specimens across different analytical platforms. Such studies would allow the development of analyte-specific reporting and rejection thresholds that balance analytical reliability with the practical difficulties of repeat blood collection in children.

## 5. Conclusions

Hemolysis produced analyte-dependent changes in the activities of the investigated plasma enzymes, with the magnitude and direction of interference varying according to the enzyme and the degree of hemolysis. LDH and AST were the most strongly affected parameters, showing substantial increases even at the lowest degree of hemolysis, while CK also demonstrated predominantly positive interference. ALT showed smaller increases, particularly at lower hemolysis levels, whereas AMY and ALP exhibited modest negative bias. GGT showed no consistent relationship with hemolysis severity. These findings indicate that even mild hemolysis may compromise the interpretation of selected enzyme activities measured in pediatric lithium-heparin plasma and emphasize the need for analyte-specific, method-specific, and matrix-specific evaluation of hemolyzed samples. The observed H1–H5 effects should therefore not be interpreted as universal quantitative thresholds for result reporting or sample rejection.

## Figures and Tables

**Table 1:** Mean percentage bias in enzyme activities and potassium concentration relative to the corresponding untreated reference aliquots.

**Analyte**	**H1, n = 22**	**H2, n = 14**	**H3, n = 10**	**H4, n = 10**	**H5, n = 9**
AST	+37.94	+68.97	+85.55	+125.84	+234.27
ALT	+3.44	+9.17	+10.17	+23.68	+35.46
LDH	+90.44	+150.17	+215.71	+319.92	+480.28
CK	+7.05	+24.70	+16.00	+34.19	+48.66
K^+^	+22.46	+33.26	+34.70	+54.26	+70.73
GGT	−0.95	−7.09	−2.67	+2.24	−9.66
AMY	−1.99	−4.35	−5.03	−6.55	−8.49
ALP	−1.25	−3.28	−3.75	−5.38	−8.99

Values are expressed as the mean percentage bias relative to the corresponding untreated reference aliquot obtained from the same original specimen. Positive values indicate increased measurements, whereas negative values indicate decreased measurements.

**Table 2:** Absolute results in untreated reference aliquots and the corresponding H1 and H5 hemolyzed aliquots.

Analyte	Unit	Baseline for H1 Pairs, n = 22, Median (IQR)	H1, n = 22, Median (IQR)	Baseline for H5 Pairs, n = 9, Median (IQR)	H5, n = 9, Median (IQR)
AST	µkat/L	0.44 (0.35–0.55)	0.60 (0.47–0.75)	0.47 (0.39–0.59)	1.52 (1.18–1.93)
ALT	µkat/L	0.34 (0.27–0.43)	0.35 (0.28–0.45)	0.36 (0.29–0.45)	0.48 (0.37–0.60)
LDH	µkat/L	3.35 (2.75–4.05)	6.10 (5.15–7.55)	3.55 (3.00–4.30)	19.10 (15.60–23.90)
CK	µkat/L	1.18 (0.80–1.72)	1.25 (0.86–1.82)	1.26 (0.88–1.85)	1.84 (1.24–2.65)
K^+^	mmol/L	4.08 (3.88–4.33)	4.96 (4.66–5.40)	4.12 (3.94–4.38)	7.05 (6.48–7.72)
GGT	µkat/L	0.23 (0.16–0.31)	0.23 (0.15–0.30)	0.22 (0.15–0.30)	0.20 (0.14–0.28)
AMY	µkat/L	1.06 (0.79–1.32)	1.03 (0.76–1.29)	1.04 (0.76–1.29)	0.95 (0.69–1.19)
ALP	µkat/L	4.55 (3.62–5.54)	4.49 (3.55–5.46)	4.62 (3.70–5.61)	4.21 (3.31–5.12)

**Table 3:** Raw and Holm-adjusted *p*-values for paired comparisons.

Analyte	H1 Raw/Adjusted	H2 Raw/Adjusted	H3 Raw/Adjusted	H4 Raw/Adjusted	H5 Raw/Adjusted
AST	<0.001/<0.001	0.002/0.007	0.010/0.016	0.004/0.012	0.008/0.016
ALT	0.004/0.020	0.032/0.064	0.105/0.105	0.006/0.024	0.008/0.024
LDH	<0.001/<0.001	<0.001/0.001	0.002/0.006	0.006/0.008	0.004/0.008
CK	0.004/0.009	0.002/0.008	0.007/0.009	0.001/0.005	0.003/0.009
K^+^	<0.001/0.001	0.003/0.012	0.014/0.023	0.006/0.018	0.012/0.023
GGT	0.602/1.000	0.094/0.282	0.043/0.172	0.711/1.000	0.031/0.155
AMY	0.214/0.214	0.028/0.112	0.034/0.112	0.008/0.040	0.041/0.112
ALP	0.071/0.284	0.181/0.543	0.238/0.543	0.009/0.045	0.412/0.543

Raw *p*-values were obtained using the paired Wilcoxon signed-rank test. Adjustment was performed separately for the five hemolysis-category comparisons within each analyte using the Holm procedure.

## Data Availability

Data available upon reasonable request.

## Abbreviations

The following abbreviations are used in this manuscript:

ALP	Alkaline phosphatase
ALT	Alanine aminotransferase
AMY	Alpha-amylase
AST	Aspartate aminotransferase
CK	Creatine kinase
GGT	Gamma-glutamyl transferase
H1–H5	Hemolysis categories 1–5
IFCC	International Federation of Clinical Chemistry and Laboratory Medicine
IQR	Interquartile range
LDH	Lactate dehydrogenase
UV	Ultraviolet
